# MicroRNAs in Barrett's esophagus: future prospects

**DOI:** 10.3389/fgene.2014.00069

**Published:** 2014-04-03

**Authors:** Juntaro Matsuzaki, Hidekazu Suzuki

**Affiliations:** ^1^Center for Preventive Medicine, Keio University HospitalTokyo, Japan; ^2^Division of Gastroenterology and Hepatology, Department of Internal Medicine, Keio University School of MedicineTokyo, Japan

**Keywords:** Barrett's esophagus, esophageal adenocarcinoma, microRNA, biomarkers, prediction

Esophageal adenocarcinoma is an aggressive malignancy with a poor prognosis. In Western countries, the incidence of esophageal adenocarcinoma has increased dramatically in the last three decades. To improve patient survival and reduce disease burden, early-stage detection, or better yet, preventing the progression of esophageal adenocarcinoma from its premalignant lesions, constitute the best short-term options. Barrett's esophagus is histologically characterized by the replacement of the normal stratified squamous epithelium of the esophagus with a columnar epithelium with intestinal differentiation (Matsuzaki et al., 2010, 2011). Barrett's esophagus is considered to be a complication of gastroesophageal reflux disease and a precursor lesion of esophageal adenocarcinoma. It is generally believed that the progression of Barrett's esophagus involves a series of histological changes: non-dysplastic Barrett's metaplasia, low-grade dysplasia, high-grade dysplasia, and ultimately, adenocarcinoma. Although these features justify endoscopic surveillance for the premalignant stages, patients with Barrett's esophagus show an absolute annual risk of only 0.12% for the development of esophageal adenocarcinoma (Hvid-Jensen et al., 2011). Therefore, recommending the invasive and expensive conventional endoscopic screening procedure is deemed controversial. In fact, Corley et al. reported that, within a large community-based population, endoscopic surveillance of Barrett's esophagus was not associated with a substantial decrease in the risk of death from esophageal adenocarcinoma (Corley et al., 2013). Thus, identification of better risk stratification biomarkers to determine the risk of progression from Barrett's esophagus to esophageal adenocarcinoma may improve disease outcome and make patient management more cost-efficient.

MicroRNAs (miRNAs) are a class of small non-coding endogenous RNAs, 18–25 nucleotides in length, and are capable of simultaneous regulation of genes by binding to target mRNAs, resulting in mRNA degradation or translational inhibition. miRNAs participate in many essential biological processes, including proliferation, differentiation, apoptosis, necrosis, autophagy, and stress responses (Saito et al., 2011b, 2012a). miRNAs have also been shown to play a potential role in cancer pathogenesis through their functions as oncogenes or tumor suppressors, depending on their gene targets (Saito et al., 2009a, 2011a; Nishizawa and Suzuki, 2013). Compared to mRNAs, miRNAs are less numerous in humans and have been proposed to act as better biomarkers by virtue of their small size, greater stability, and capability of regulating hundreds of mRNAs. Therefore, miRNAs profiling could improve the risk stratification for the progression of Barrett's esophagus to esophageal adenocarcinoma.

MiRNAs can be profiled on a genome-wide scale using array or sequencing technologies. However, very few studies have been conducted to identify miRNAs as prognostic biomarkers for the progression of Barrett's esophagus to adenocarcinoma. Although several cross-sectional studies using comprehensive array analysis have been reported (Feber et al., 2008; Kan et al., 2009; Yang et al., 2009; Fassan et al., 2011; Leidner et al., 2012; Wu et al., 2013), their results have proved controversial. They compared the expression of miRNAs across different types of histological specimens such as Barrett's esophagus, low-grade dysplasia, high-grade dysplasia, and esophageal adenocarcinoma, and reported that a substantial number of miRNAs show differential expression in esophageal tissues (Sakai et al., 2013). Indeed, they might be useful in revealing certain mechanisms underlying carcinogenesis. But, they might be difficult to identify risk stratification biomarkers. We should think about much better research strategies.

Recently, two nice studies were reported to identify risk stratification biomarkers for Barrett's esophagus: one prospective study and one cross-sectional study. First, Revilla-Nuin et al. have reported a set of miRNAs associated with this progression and provided further validation in two groups of patients with Barrett's esophagus, who either developed or did not develop adenocarcinoma, over a course of 5 years (Revilla-Nuin et al., 2013). Among 24 patients with Barrett's esophagus, 7 patients progressed to adenocarcinoma while the other 17 did not. Four miRNAs (*miR-192*, *miR-194*, *miR-196a*, and *miR-196b*) were found to show significantly higher expression in patients with progression to esophageal adenocarcinoma than in patients who did not show disease progression. Second, Saad et al. conducted a notable comprehensive microarray profiling for identifying the specific miRNA signature associated with esophageal adenocarcinoma (Saad et al., 2013). They analyzed 13 samples from isolated Barrett's esophagus, 10 from Barrett's esophagus adjacent to high-grade dysplasia, 17 from high-grade dysplasia, and 34 from esophageal adenocarcinoma tissue. They identified that *miR-21*, *miR-31*, *miR-192*, and *miR-194* were upregulated in Barrett's esophagus adjacent to high-grade dysplasia lesions as compared to isolated Barrett' esophagus. In addition, these 4 miRNAs were upregulated in a progressive manner through the Barrett's metaplasia-dysplasia-adenocarcinoma sequence. More importantly, this study provided findings for Barrett's esophagus for two groups: isolated Barrett's esophagus vs. Barrett's esophagus adjacent to high-grade dysplasia. The limitations of both two papers include the very small sample size. Larger prospective multi-institutional studies are warranted to confirm this result. Another criticism against the studies using comprehensive microarray analysis is that these could not provide the insights how miRNAs may exert their effects (Saito et al., 2009b, 2012b, 2013).

Since clinical predictors of increased risk of esophageal adenocarcinoma, namely, the length of Barrett's esophagus, male gender, older age, current tobacco smoking, alcohol consumption, central obesity, and bile reflux, have been established, the association between the expression levels of miRNA in Barrett's esophagus and these clinical risk factors would require further investigation. We had recently reported that expression levels of *miR-221* and *miR-222* increased when cultured esophageal epithelial cells were exposed to bile acids. *miR-221* and *miR-222* are known to specifically target p27Kip1, which in turn inhibits the proteasomal protein degradation of CDX2 (caudal-related homolog 2) (Matsuzaki et al., 2013). Furthermore, *miR-221* and *miR-222* expressions are higher in esophageal adenocarcinoma than in the surrounding Barrett's esophagus. We also confirmed that the levels of p27Kip1 and CDX2 were lower in areas of esophageal adenocarcinoma than in those of Barrett's esophagus. Thus, we showed that the degradation of CDX2 was enhanced by upregulation of *miR-221* and *miR-222* on exposure to bile acids. Although bile acids are known to induce DNA damage, resistance to apoptosis through NF-κB activation, and resistance to autophagy (Fang et al., 2013), the association between bile acids and miRNA expression has never been reported except for our results (Masaoka and Suzuki, 2014). In this way, clinical epidemiological information would be important and useful to reveal novel insights of miRNA in the progression of Barrett's esophagus to adenocarcinoma.

In conclusion, on the basis of clinical importance, better risk stratification biomarkers to determine the risk of progression from Barrett's esophagus to esophageal adenocarcinoma are expected. We should deepen our knowledge of miRNA using clinical materials, hopefully with more prospective approach. The fusion of basic science and clinical science research would also be required for identifying the upstream regulation and the downstream targets of miRNAs and understanding their mode of action. These will facilitate the development of miRNA-based prevention or therapeutic strategies for esophageal adenocarcinoma.

## Conflict of interest statement

The authors declare that the research was conducted in the absence of any commercial or financial relationships that could be construed as a potential conflict of interest.
